# A qualitative study of the perception of nursing home practitioners about the implementation of quality indicators for drug consumption in nursing homes

**DOI:** 10.1007/s40520-021-01989-7

**Published:** 2021-10-06

**Authors:** Stéphane Sanchez, Fiona Ecarnot, Dimitri Voilmy, Biné Mariam Ndiongue, Clément Cormi, Aude Letty, Paul Emile Hay, Jean Luc Novella

**Affiliations:** 1 Hôpitaux Champagne Sud – Centre Hospitalier de Troyes, Troyes, France; 2 Fondation Korian Pour Le Bien Vieillir, Paris, France; 3 Cardiology Department, University Hospital Besancon, Boulevard Fleming, 25000 Besancon, France; 4 EA3920, University of Franche-Comté, 25000 Besançon, France; 5 Living Lab ActivAgeing, UTT de Troyes, Troyes, France; 6 Service de Gériatrie, CHU de Reims, Reims, France; 7 EA3797, University of Reims Champagne Ardennes, Reims, France

**Keywords:** Qualitative study, Focus groups, Deprescribing, Nursing homes

## Abstract

**Introduction:**

Nursing homes (NHs) are an ideal environment in which to implement interventions aimed at reducing inappropriate prescriptions. Quality indicators (QIs) may be useful to standardize practices, but it is unclear how they mediate change. In the framework of a quantitative study aimed at reducing the prescription of anticholinergic drugs among NH residents using QIs, we performed a qualitative study to describe the investigators’ perception of the utility of QIs.

**Methods:**

Qualitative study using focus group methodology. Focus groups were recorded and transcribed, and analyzed by thematic analysis. Participants were purposefully recruited from among the medical directors of the NHs in the quantitative study.

**Results:**

Five medical directors participated in two focus group meetings. The main themes to emerge were: (1) communication is key to introducing new practices and achieving lasting uptake; (2) improved coordination and communication provided useful information to help interpret the quantitative results observed: e.g., participants reported that they were able to obtain contextual and patient-specific information that explained why some prescribers had consistently, but justifiably “poor” performance on the quantitative indicators; (3) negative aspects reported included reluctance to change among prescribers and the tendency to shirk responsibility.

**Conclusion:**

From the point of view of medical directors of NHs participating in an interventional program to reduce inappropriate prescriptions of anticholinergic drugs, the main factor driving the success of the program was communication, which is key to achieving adherence. Improved communication provides useful insights into the reasons why no quantitative reduction is observed in objective quality indicators.

## Background

Nursing home residents are often exposed to polypharmacy. Therefore, nursing homes are an ideal environment in which to implement interventions aimed at reducing inappropriate prescriptions and preventing medication misadventure. Various strategies have been tested, with varying degrees of success, to optimize medical prescriptions for nursing home residents, including specific training for physicians, collaborative initiatives between physicians and pharmacists, collaborative medication review, or pharmacist-led medication reconciliation [1–3].

In France, nursing home residents can choose their own general practitioner (GP), with the result that many GPs may be prescribing for a single nursing home, generating considerable heterogeneity among prescriptions. Indeed, although nursing homes in France have the particularity of employing a medical director and a director of nursing, neither of these professionals can prescribe medications for the residents. They are responsible for overall coordination of care for the nursing home, navigating among the many health professionals who are in contact with the residents.

Across this complex network of healthcare professionals, it can be difficult to achieve standardized practices. In this regard, feedback, audits and quality indicators can be useful in improving the quality of care [4, 5]. Feedback improves quality by providing an objective metric assessing individual and/or collective practices, and incites those evaluated to change their practice to improve their performance [6]. Quality indicators, audit and feedback may also motivate change through social pressure and a competitive edge. Yet, studies demonstrating the efficacy of these metrics in bringing about positive change fail to identify the exact aspects of the intervention, or the precise characteristics of the environment in which the intervention was implemented that drive the improvement in practices [7].

To this end, in the framework of a quantitative study implemented by our group aimed at reducing the prescription of drugs with anticholinergic properties among nursing home residents through the use of quality indicators, we performed a qualitative study using focus group methodology to investigate, among participating investigators in the quantitative study, their perception of the study and the use of quality indicators, with a view to identifying the factors driving the success of the program. This would enable us to ensure the reproducibility of the intervention on a larger scale.

## Methods

In the context of the quasi-experimental quantitative DEMASCH study, we conducted the present qualitative study using focus group methodology in a mixed methods outlook. Briefly, the quantitative DESMASCH study aimed to assess the impact on the prescription of drugs with anticholinergic properties, of an intervention designed to improve prescribing practices with respect to this particular drug class. The intervention comprised educational sessions and dedicated materials for nursing home staff (unlimited access to study material for staff, including nurses, general practitioners, and pharmacists). Furthermore, lists of alternative drugs were provided that could be used as substitutes and regular feedback was provided using quality indicators to participating nursing homes to track their progress in terms of anticholinergic prescriptions over a period of 18 months. Indicators were calculated based on routine data collected from an electronic pill dispenser system in the participating nursing homes, and the primary outcome of the quantitative study was the presence of at least one prescription containing ≥ 1 drug from among a list of 12 drugs with anticholinergic properties. There were four quality indicators automatically calculated from routine prescription data (namely, the overall proportion of patients receiving anticholinergics; the number of patients who received at one least 1 drug with anticholinergic properties and among these patients, the total number of drugs with anticholinergic properties per patient; and the average number of drug prescriptions per patient). The indicators were sent monthly to the nursing home medical director who shared it with prescribing physicians during the study period.

In parallel, the present qualitative study using focus group methodology was conducted. Focus groups were chosen as a means of collecting data through moderated group discussions that would provide participants’ personal perceptions and experience of the ongoing quantitative study and help inform our understanding of the quantitative results observed. Given that the quantitative DEMASCH study was investigating the capacity of an educational intervention to durably change practices, we hypothesized that acceptance by all the stakeholders would be crucial to bringing about lasting change. In addition, collaboration and communication, which are key features of focus groups, were also hypothesized to be necessary foundations for lasting changes in prescription practices to be achieved.

### Data collection

Participants were purposefully recruited from among the medical directors of the nursing homes in the intervention group of the quantitative DEMASCH study (*n* = 10 directors). Five medical directors participated in both focus group sessions. The other five were unable to participate due to organizational constraints (unavailable at the time of the focus groups). All participants provided written informed consent. Participants did not receive any compensation or incentive. The focus groups were organized in June and September 2019. The first focus group lasted 1 h and 16 min, and the second lasted 40 min.

The discussions were audio-recorded and professionally transcribed for later analysis and translation. Transcripts were not returned to participants for comments or corrections. The focus groups were led by researchers experienced in facilitating focus groups, and who took notes during the focus group (in addition to the recording). The facilitators used a brief interview guide to ensure that all the relevant areas were covered. The interview guide was developed in collaboration with the study research team (methodologists, geriatric medicine specialists) based on a review of the literature. The facilitators strove to create an informal and open atmosphere that would enable participants to share their experience and exchange ideas between themselves.

The guide covered the following points:

1. What was your perception of the DEMASCH study?
2. What do you think about the efficacy of the study?
3. How did you manage the practical implementation, maintaining motivation over the study duration and acceptance of the study by prescribers?
4. What impact did the project have on your relations with prescribers?
5. What impact did the project have on your relations with healthcare teams and pharmacists?
6. What about continuing this project and/or extending it to other molecules/therapeutic classes?
7. Did you learn anything from your experience of participating in the DEMASCH study?

### Methods of analysis

The discussion was transcribed verbatim, and thematic analysis was performed on the French transcripts prior to translation. During transcription, and with the participant’s permission, first names were left unchanged. However, the transcriptions were strictly confidential and shared only with the researchers involved in the analysis. Four researchers [SS (male, medical doctor), DV (male, PhD in sociology; social science research), FE (female, PhD in qualitative research and specific training in grounded theory; clinical researcher)] independently read the transcripts and performed thematic analysis [8, 9]. First, transcripts were coded with open coding. Then, recurring themes were classed into major and minor themes. Major themes are points that are mentioned spontaneously by several participants and discussed at length. Minor themes are points that are mentioned by some, but not all participants, and discussed in less detail. The themes were then examined for interrelations and a schematic framework of the main ideas was developed. NVivo software was used to assist with data management (QSR International Pty Ltd.).

This study is reported in compliance with the COnsolidated criteria for REporting Qualitative guidelines, a 32-item checklist used in the reporting of data from qualitative research (including interviews and focus groups) [10].

## Results

**Fig. 1:**
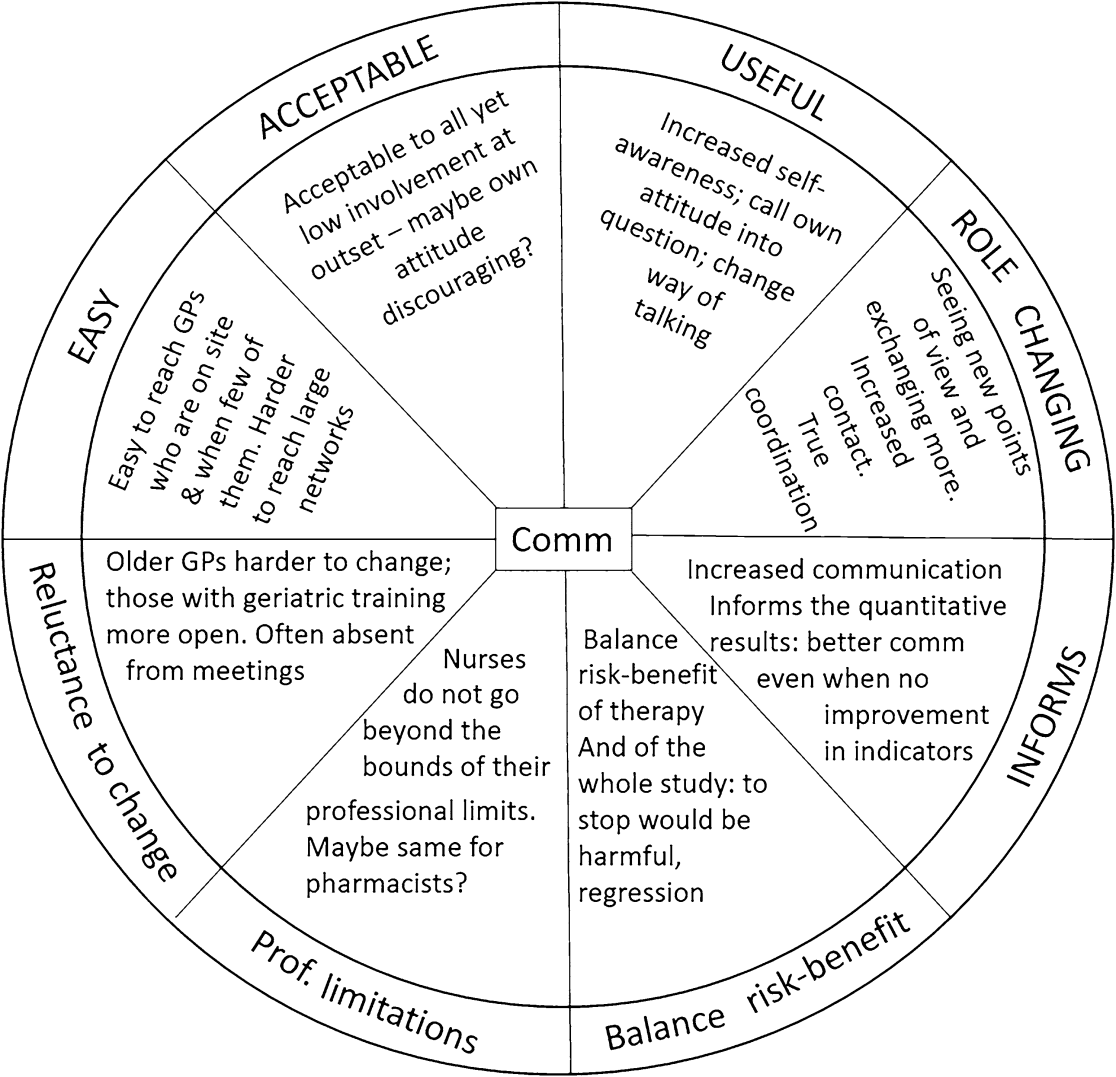
Main themes emerging from the qualitative analysis. *Comm* communication; *Prof* professional; *GP* general practitioner

A schematic representation of the main findings is presented in Fig. 1.

1. *Communication is key*
  Overall, the central point to emerge is that communication is key to introducing new practices and achieving lasting uptake. At the outset, the focus group participants found that the study seemed to be generally easy to implement and acceptable to the prescribers. In terms of ease of implementation, the participating medical directors reported that it was easy for them to contact GPs who had their medical practice closeby, or within the nursing home. This geographical proximity meant firstly that contacts were more frequent, and secondly that there was already an existing working relationship that made it easy to broach the subject of new prescribing practices.
  The participants also reported that when they sought to introduce the intervention, they found it was generally acceptable to the prescribers involved, but this superficial enthusiasm did not necessarily translate into a strong level of implication. In this regard, when relating this experience, it dawned on some of the participants that their own manner of presenting the intervention may have influenced the participation, because they may have (likely unintentionally) appeared skeptical themselves. This realization had the advantage of making the focus group participants reflect on their own manner of expressing themselves and addressing others. For example, instead of telling the GPs that “what you’re doing is not appropriate”, they came to the conclusion that it is more productive to approach them with an inclusive question, like “Don’t you think that we could this better?”
  In addition to being acceptable, all the study participants reported that the intervention was perceived as being useful. For themselves, it increased their self-awareness about their attitude to change and their manner of expressing themselves and addressing other colleagues. It also helped them to see new points of view, for example through the eyes of the prescribers prescribing for their nursing home residents, and it improved communication with these prescribers. This is essential for the medical director, whose role is strategic and focused on coordination. Therefore, the act of approaching their prescribers, introducing this intervention, and following up with those prescribers regarding their performance indicators truly aligns with the coordinating role of the medical director.
2. *Improved communication informs interpretation of quantitative results*
  This improved coordination and communication provided useful information to help interpret the results observed in the quantitative study. Indeed, some prescribers had consistently high rates of prescription of anticholinergic drugs, and so their performance indicators remained “in the red”. However, the medical directors were able to personally contact the prescribers and obtain more information about the context. So, for example, it became clear that some patients were on drug regimens that had taken a long time to be stabilized in collaboration with a range of other health professionals and could therefore not be changed. Or, some patients had drug regimens prescribed by other establishments for specific medical problems, and maintenance of those drugs was justified. In this way, even though no quantitative reduction was obtained in the prescription of anticholinergics by certain prescribers, the focus group discussions brought to light the fact that the use of performance indicators opened the door to discussions. By enabling discussion and improving communication, there was indeed a qualitative benefit, despite the absence of a quantitative improvement in the performance indicators. This in itself represents an improvement in appropriate practice.
3. *Negative aspects of the study: reluctance to change and shirking responsibility*
  Moving on from the benefits and positive points, the participants also mentioned some more negative aspects encountered. The risk–benefit ratio of the drugs being prescribed needed to be evaluated, which was beyond the scope of the medical directors participating in our focus groups, since they were not the prescribers. They felt nonetheless that once such an initiative to reduce inappropriate prescriptions is undertaken, it should not be discontinued, because that would represent a potential regression. Another practical limitation reported by the medical directors participating in the focus groups was the level of involvement of each of the different groups involved in the quantitative study, notably the tendency of each group to shirk responsibility, and say that it is incumbent on others. For example, the pharmacists contacted about the intervention were, for the most part, not heavily involved, often citing the fact that it is not their role to contradict the prescribing GP, especially when the pharmacist has no knowledge of the patient’s context or symptoms. Similarly, the nurses in the nursing homes were initially enthusiastic, but in the end they cannot go beyond the limits of their profession, and while they can alert the prescribing physicians to the presence of symptoms, they cannot, themselves, change the prescriptions. Finally, the prescribers (GPs) are often reluctant to change their habits. Younger physicians seemed to be more receptive to the use of technological aids for prescription and for obtaining information. There was also a certain unwillingness among many GPs to change a prescription made by a colleague in another establishment or medical practice. Many of these GPs could be classed as “hard to reach” targets in whom durable change might be difficult to achieve.

### Proposals for good practice with a view to achieving a lasting change

The results of these focus groups provide insights into the aspects of an intervention that are most difficult to implement, as well as the aspects that are most helpful in effecting lasting changes in behavior. We thus propose the following pointers, based on these results, as a basis for interventions aimed at changing practices in a large group of disparate medical professionals:

- Dialogue is key: propose, do not impose.
- Discussing a specific patient file or case can be enlightening about overall attitudes.
- GPs are generally willing to listen to coordinators and take suggestions on board.
- Prefer in-person exchange, where possible, over emails or phone calls.
- Holding focus groups with the population being targeted by the intervention (e.g., GPs) can bring about self-evaluation and improved self-awareness through the collective intelligence generated in a group discussion.

## Discussion

The results of this qualitative study show that an intervention aimed at reducing inappropriate prescriptions in nursing homes can be properly implemented and is well accepted by the numerous professionals that are involved in the care processes for the residents. From the point of view of the medical directors of the participating nursing homes, the main factors to emerge is that communication is key to achieving adherence and to understanding the results obtained in terms of quality indicators. Indeed, communication may provide useful insights to explain why no quantitative reduction is observed, which in itself represents a qualitative result. These findings underline the importance of communication between healthcare providers in preventing polypharmacy in the nursing home setting. Our findings are also coherent with quantitative studies showing the efficacy of multidisciplinary interventions bringing together professionals from various specialties [11, 12].

In parallel, the importance of some of the professionals may have been underestimated by our participants. In particular, from our focus group transcripts, it did not appear that the role of nurses was preponderant in adjusting patients’ treatment. The medical directors did express the sentiment that the nurses have a role in reporting patient symptoms, but that it was beyond the scope of their professional mandate to suggest or order changes to prescriptions. In a qualitative study investigating the perspectives of patients, informal caregivers, nurses and physicians on the role of nurses in medication management at the end of life, it was found that nurses have a particularly important role in the continuity of care and proximity to the patient, making them a valuable intermediary between physician and the patient [13]. Our findings are in line with this, but suggest that increased consideration of the nurses’ opinions by the prescribing physicians may be helpful when reviewing medical prescriptions.

Participants in our study were unanimous on several points. Firstly, they all agreed on the utility of pursuing this initiative beyond the official end of the quantitative study, despite the difficulties they encountered in implementing the intervention. They all felt that the study was truly useful, and the minor “failures” they faced would not discourage them from pursuing or extending the initiative. This is coherent with previous reports in the literature. In a qualitative study regarding deprescribing in Swiss nursing homes, Foley et al. reported that at an individual level, the different healthcare professionals had concerns about deprescribing that were shaped by their specific role within the nursing home [14]. Nevertheless, their perspective about the different means of promoting deprescribing at the level of the institution and healthcare system converged toward interprofessional collaboration, supported by the context of healthcare delivery. In particular, Foley et al. underlined that specific funding and incentives are essential to support a sustainable interprofessional initiative [14]. Clearly, in such a context, the means to leverage prescribing practices do not depend solely on sporadic initiatives or educational interventions, but also require a favorable healthcare system that is amenable to developing and sustaining such initiatives with funding.

Our participants reported that for the residents, there is a real advantage to be gained from the improved communication brought about by the study intervention. Indeed, improved communication between all the patients’ healthcare providers, in conjunction with improved communication with the patient themselves, ultimately leads to greater adherence and satisfaction. This has previously been described in the literature, in a study showing that contrary to popular belief, nursing home residents and their relatives are not reluctant to consider a treatment reduction, but rather would be more than willing to consider it, provided that time is invested to explain the expected benefits of such changes to them [15]. This concept is congruent with the recommendations of Muller et al. regarding measures to improve interprofessional collaboration and communication [16]. Developed iteratively in a continuous process involving nine focus groups of GPs and nurses, Muller et al. identified six measures to improve collaboration and communication, namely: meetings to establish common goals, designating a main contact person, standardized medication, introduction of name badges, improved availability of nurse/GP and standardized scheduling/procedures for visits [16]. They reported that the measures were feasible and acceptable, with only the designation of a main contact person failing to be of any practical improvement. The intervention implemented in the nursing homes of our study participants largely coincided with these measures, and the findings of the present qualitative study show that, indeed, the intervention prompted improved communication between professionals (both formal and informal), which was felt to be truly beneficial. By necessity, discussion between several professionals about a specific patient amounts to defining common goals, since the purpose of such a discussion is to come to an agreement about the best therapeutic strategy to pursue. Therefore, the present study reveals that in practice, interprofessional communication is a key element driving the success of the intervention. The collaborative exchange, defining common goals for each patient, can help to achieve the objective of improved prescription practice, without the prescribers perceiving the need for a reduction in prescriptions as a constraint. This in turn contributes to greater acceptability of the project.

Finally, the use of objective quality indicators in the quantitative DEMASCH study was perceived by the medical directors as a useful means to generate a healthy degree of competition between practitioners and prompt them to strive to achieve excellence. It has previously been shown among nurses participating in a quality improvement initiative in Swedish nursing homes that doing good work for the benefit of the residents was strongly connected to a sense of professional pride [17]. The participants in our focus groups were in agreement that the intervention helped to stimulate professionals to do better by giving them feelings of pride when their indicators were good, while improved communication helped to understand why the indicators of some professionals did not numerically improve, without stigmatizing them for “poor performance”.

### Study limitations

The originality of the present study is that it investigates the perceptions of medical directors from nursing homes about the factors that drive the successful implementation of quality improvement initiative aimed at reducing unnecessary prescription of anticholinergic medications. The findings provide insights into the factors that enable the uptake of and adherence to the project. Nevertheless, our study also has some limitations. Firstly, only medical directors of the participating nursing homes were included, even though previous studies have insisted on the importance of nurses in identifying inappropriate prescriptions [18]. The intervention that was tested in the DESMASCH quantitative study was designed to be applied by all the personnel in the nursing home, but for reasons of feasibility, only the medical directors were included, as they have the authority to oversee all healthcare providers working with the nursing home.

## Conclusion

From the point of view of medical directors of nursing homes participating in an interventional program to reduce the inappropriate prescription of anticholinergic drugs, the main factor that drives the success of the program is communication, which is key to achieving adherence. Furthermore, improved communication provides useful insights into the reasons why no quantitative reduction is observed in objective quality indicators in some cases. These findings underline the importance of communication between healthcare providers in preventing inappropriate prescriptions in the nursing home setting.

## Data Availability

The data and materials supporting the results of this study are available from the lead investigator (SS) on reasonable written request and in their original language.

## References

[1] Alldred DP, Kennedy MC, Hughes C (2016). Interventions to optimise prescribing for older people in care homes. Cochrane Database Syst Rev.

[2] Cancelli I, Beltrame M, Gigli GL (2009). Drugs with anticholinergic properties: cognitive and neuropsychiatric side-effects in elderly patients. Neurol Sci.

[3] Crotty M, Halbert J, Rowett D (2004). An outreach geriatric medication advisory service in residential aged care: a randomised controlled trial of case conferencing. Age Ageing.

[4] Hemmila MR, Cain-Nielsen AH, Jakubus JL, Mikhail JN, Dimick JB (2018). Association of hospital participation in a regional trauma quality improvement collaborative with patient outcomes. JAMA Surg.

[5] de Vos M, Graafmans W, Kooistra M (2009). Using quality indicators to improve hospital care: a review of the literature. Int J Qual Health Care.

[6] Buntinx F, Winkens R, Grol R (1993). Influencing diagnostic and preventive performance in ambulatory care by feedback and reminders. Rev Fam Pract.

[7] Ivers NM, Sales A, Colquhoun H (2014). No more 'business as usual' with audit and feedback interventions: towards an agenda for a reinvigorated intervention. Implement Sci.

[8] Chahraoui K, Laurent A, Bioy A (2015). Psychological experience of patients 3 months after a stay in the intensive care unit: a descriptive and qualitative study. J Crit Care.

[9] Meunier-Beillard N, Ecarnot F (2017). Can qualitative research play a role in answering ethical questions in intensive care?. Ann Transl Med.

[10] Tong A, Sainsbury P, Craig J (2007). Consolidated criteria for reporting qualitative research (COREQ): a 32-item checklist for interviews and focus groups. Int J Qual Health Care.

[11] Garland CT, Guenette L, Kroger E (2021). A new care model reduces polypharmacy and potentially inappropriate medications in long-term care. J Am Med Dir Assoc.

[12] Dellinger JK, Pitzer S, Schaffler-Schaden D (2020). Improving medication appropriateness in nursing homes via structured interprofessional medication-review supported by health information technology: a non-randomized controlled study. BMC Geriatr.

[13] Huisman BAA, Geijteman ECT, Dees MK (2020). Role of nurses in medication management at the end of life: a qualitative interview study. BMC Palliat Care.

[14] Foley RA, Hurard LL, Cateau D (2020). Physicians, nurses and pharmacists perceptions of determinants to deprescribing in nursing homes considering three levels of action: a qualitative study. Pharmacy (Basel).

[15] Cateau D, Foley RA, Niquille A (2020). Deprescribing in nursing homes: comparative views of residents, their relatives, and healthcare professionals. Rev Med Suisse.

[16] Muller CA, Fleischmann N, Cavazzini C (2018). Interprofessional collaboration in nursing homes (interprof): development and piloting of measures to improve interprofessional collaboration and communication: a qualitative multicentre study. BMC Fam Pract.

[17] Vikstrom S, Johansson K (2019). Professional pride: A qualitative descriptive study of nursing home staff's experiences of how a quality development project influenced their work. J Clin Nurs.

[18] Vogelsmeier A, Anderson RA, Anbari A (2017). A qualitative study describing nursing home nurses sensemaking to detect medication order discrepancies. BMC Health Serv Res.

